# Tuberculosis and the Effectiveness of the Revised National Tuberculosis Control Program (RNTCP) to Control Tuberculosis: A Narrative Review

**DOI:** 10.7759/cureus.51418

**Published:** 2023-12-31

**Authors:** Shraddha Malwe, Dushyant Bawiskar, Vasant Wagh

**Affiliations:** 1 Community Medicine, Jawaharlal Nehru Medical College, Datta Meghe Institute of Higher Education and Research, Wardha, IND; 2 Sports Medicine, Abhinav Bindra Sports Medicine and Research Institute, Bhubaneswar, IND

**Keywords:** schachepheth, tabes, phthisis, white plague, consumption, mycobacterium tuberculosis

## Abstract

The revised National Tuberculosis (TB) Control Program is an initiative undertaken by the government of India and was active from 1997 to 2020. Later it was renamed as National TB Elimination Program, which eyes the complete eradication of TB by 2025. The revised National Tuberculosis Control Programme (RNTCP) is preceded by the National TB Control Program which was activated when the cases of TB were on the rise in the early 1960s and police intervention was needed. National Tobacco Control Cell (NTCP) guided the efforts until 1997 when various shortcomings, which were registered over the course of time, were addressed and the revised program was launched. It has been a mixed success as beneficiaries belonging to the reachable, urban areas were benefitted, and tribal, and backward areas were lagging behind. Although the RNTCP proved to be effective in containing TB and curing it to a certain extent, the successor of the program, which is NTEP, has set an ambitious goal of eradicating TB by 2025 which needs concerted efforts on behalf of all stakeholders.

## Introduction and background

Mycobacterium tuberculosis, or TB, as it is popularly called, is a pulmonary ailment usually chronic in nature. It is categorized as an infectious disease generally caused by the said bacteria that is mycobacterium tuberculosis. It is a general misconception that TB is only limited to the lungs and pulmonary functions of the body, but it can affect other parts of the body as well. The fraction of latent TB that is no presentation of the symptoms by the affected person is more and thus early diagnosis can decide the course of the treatment as well as clinical outcome [1-3]. Around one-tenth of these latent infected individuals evolved into active and symptomatic cases and if they do not seek medical intervention in time, can obtain fatal clinical outcomes in about half of those cases [4]. General symptoms involved in TB are chronic cough, spitting of blood-ridden mucus, fever, unexplained weight loss, and so on. The spread of the disease happens through bodily discharges via cough, cold, sneeze, etc. However, the latent infected individuals have less chance of spreading it as they do not show any of the symptoms. In almost 90 percent of the cases, TB is of pulmonary type that involves the lungs. Sputum containing prolonged cough is the primary sign of it. Usually three weeks after coughing starts, one should be concerned about it and seek medical intervention as soon as possible [5-7].

The diagnostics involved X-rays of the chest area and culture examination of bodily discharges. If the disease persists long enough, the upper lungs are scarred, and the pulmonary functions are severely affected. Around 15 percent of the cases are of the extrapulmonary type and can spread to other parts of the organ. Young children and individuals undergoing an immunosuppressive state are generally targets of extrapulmonary TB. Coupled with HIV, the occurrence of the cases is high as the human immunodeficiency virus is notorious for weakening the immune system of the body to extremely low levels. It can affect the pleural cavity, central nervous system, lymphatic system, genitourinary system, and bones along with joints among others besides the lungs. The risk of transmission of the disease is high particularly among the close contacts of the infected individuals as each sneeze releases 40000 droplets that contain the said bacteria in large numbers. The risk of prolonged or close contact can increase by 22 percent. Other factors that can determine the risks are viral load, time of exposure to it, the immune system of a person, and so on. Simply segregating the infected individual and putting him or her on an anti-TB drug regimen can help a lot to stop the spread [8-10].

The major risk factor remains the concurrent occurrence of HIV. Others include alcoholism, diabetes mellitus, smoking, air pollution, malnutrition, genetics, and so on. Young age is also a risk factor as the immune system of children is at the developmental stage and one needs to be cautious about exposure to active TB patients [11,12].

## Review

Methodology

A literature search in English was conducted using the electronic databases PubMed, MEDLINE, Embase, Google Scholar, and ResearchGate. The search terms were “TB” or “RNTCP, “mycobacterium tuberculosis”, or “TB elimination,”. The writers’ knowledge and experience in the field supported the archiving of relevant papers. Articles that match the following criteria are included in this review: studies in English and studies from the previous 10 years are included as well. Table 1 shows a summary of some studies that we included in the review.

**Table 1:** Summary of some studies included in the review. TB: Tuberculosis

Author	Year	Type	Conclusion
Biswas et al. [13]	2020	Original Article	Disparities in target achievement of TB diagnosis and treatment among states in India have been noticed and less literate states performed worse.
Muniyandi et al. [14]	2020	Original Article	The focus was on decentralization of the health care services, and dedicated diagnostics and supervisory autonomy was percolated to the unit level to effectively deal with the spread of the T4.
Visca et al. [15]	2020	Review Article	A novel approach suggested by the report, which is essential in order to trace the patients without investing extra resources, is testing both COVID-19 and TB suspects at the same time. It will ensure timely detection.
Parmar et al. [16]	2018	Research Article	MDR-TB patients also need to be focused as the drug regimen has been less effective among those along with progressing XDR-TB [16].

Status of TB in India

The status of TB in India is grave as compared to other parts of the world. More than two million people infected with TB belong to India, of the 9.6 million people around the world. The disproportionate numbers are a serious cause of concern. The drug-resistant version of TB is making the situation even worse as the drug regimen followed while treating the TB patient is rendered ineffective in the case of drug-resistant TB. The progression of the disease was firstly from multi-drug-resistant TB to Extensively drug-resistant TB and finally to totally drug-resistant TB [17,18]. In monetary terms, the cost bore by the nation was USD $1 billion. The World Health Organization has already termed the progression of TB in India as an epidemic, which carries 26 percent of the active cases worldwide. The incidence rate in the case of India stood at 192 per 100000 population. India, which is a developing country, has a lot of socioeconomically disadvantaged people having TB which can have a negative impact on a global campaign to eliminate TB. Ten or more people can be infected by one infected individual in a year. However, the patient needs to be symptomatic, as they can spread the infection through nasal and oral discharges [10,13,19].

Revised National Tuberculosis Control Program

The revised National Tuberculosis Control Program or RNTCP was the mitigating strategy devised by the government of India to contain the spread of TB all over India. It was effective until 2020 when it was replaced by National TB Elimination Program (NTEP). Now the goal is to eliminate TB by the year 2025. It all started when the cases of TB were on the rise and whooping cough was turning into TB which really affects the quality of life of the patients. The effect on pulmonary activity has a cascading impact on another organ system as oxygen is the vital component for each and every cell of the body. In 1962, considering the increasing number of cases of TB, the government of India started a program to curb the spread called the National TB Control Program [20-22]. Its major objective was to detect the cases early enough so that they could be treated in time and save the patients from slipping to severe clinical conditions arising out of it. It primarily focused on the vaccination, BCG, as a preventive measure. International agencies like the UN and WHO were backing the initiative. It was running until 1997 when it got reorganized as RNTCP. The deficiencies like lack of proper monitoring, improper assessment of the health outcomes, and inefficient diagnostic techniques in previous programs were rectified in the RNTCP. The major difference was the shift from only preventive to preventive along with the curative strategy. It was found that after the detection of TB, the curability was high in 6 to 12 months [23-25]. A treatment-based strategy was designed to deal with the increasing number of cases. A strategy called directly observed treatment short course (DOTS) was accepted all over the world as a cost-effective as well as systematic approach to deal with the spread of TB. Previously the completion and follow-up rate for TB patients was low but after the DOTS due to its short and effective intervention along with its inexpensive nature, the completion rate is quite high. Various sub-districts have supervisory units, which are popularly known as TB units [14,26,27]. The focus was on decentralization of the health care services and dedicated diagnostics and supervisory autonomy was percolated to the unit level to effectively deal with the spread of TB. The first phase of the RNTCP spanning over eight years (1998-2005) mainly targeted the accessibility of DOTS treatment all over India. One-hundred and two districts were targeted in the first phase and another 203 districts were being conditioned for the full rollout of the plan [28]. By the year 20025, the government moved to a full-scale nationwide rollout of the RNTCP program, particularly DOTS. By March 2006, India achieved nationwide coverage of the RNTCP. The target was set to 70 percentage in terms of detection rate by smear positives and 85 percentages in cure rate. It was also coinciding with the targets set by the millennium Development Goals by 2015. Although the phase one and two of the RNTCP were almost the same the services were upgraded in phase two and the inclusion of multi-drug-resistant TB was covered under the DOTS plus program. Other aspects are often undermined but need a strategic overlook such as advocacy about getting treated post-infection, communication of the messages and campaigns which helps to ease the tense situation, and social mobilization to deal with any kind of hesitancy attached to it [29-31]. Along with demystifying, the myths associated with it are also included in phase 2 of the RNTCP. A web portal named NIKSHAY has been activated in 2012 helps to congregate the anonymized data to build insightful conclusions that will guide further efforts. After 2020, the program changes its target from control to elimination of the bacterial disease. By 2025, the government is planning to achieve the complete elimination of TB by preventing and curating it wherever necessary. RNTCP was renamed as the National TB Elimination Program (NTEP) with effect from the 1st of January 2020. Ministry of Health and Family Welfare is the nodal ministry under the government of India for the program that will guide the state as health is listed as the state subject in the Constitution of India. A policy document National Strategic Plan for Tuberculosis Control for 2017 - 2025 was released to guide the efforts of the elimination of TB [28].

Several studies have been done to assess the outcomes of the RNTCP. So far RNTCP has achieved the objectives, especially after 2007 along with globally set targets for control of the TB. The regimen of six months for TB meningitis and bones and joints under RNTCP has been questioned about its efficacy as there is a similar regimen for 9 to 12 months which has been accepted worldwide. The advocacy and social mobilization components were introduced but there are many patients who do not know about the free diagnostic and treatments which are provided under the RNTCP. They seek help from private clinics and other facilities even quacks in some cases if they are socioeconomically well. Half of the total TB patients are availing of the treatments available other than RNTCP facilities. In the case of India, merely 0.31 percent of doctors are engaged in activities under RNTCP. These private practitioners are not rained under RNTCP to treat TB and there are huge disparities between the treatment strategies offered under RNTCP and the ones provided by private practitioners [32-34]. There is a need for close collaboration with the private stakeholders as these are some of the most important factors in controlling TB. Here is also a lack of health care professionals in the public sector and this can hamper the progress made under RNTCP. This can be solved by raising parallel private healthcare professionals who are already engaged in similar activities. Medical colleges are hot spots for starting such activities and they can encourage research and outcome-oriented studies in order to study the outcomes of the program.

The socioeconomic conditions of many patients are not well enough to afford any private medical intervention. They are covered under the RNTCP program. Widespread coverage is necessary to ensure the detection of all cases, as undetected active cases with symptoms can be very problematic as they can spread the disease to around 10 more people in a year. In addition, their condition worsened as the days passed without any treatment [15,35,36]. A study to check the success rate of RNTCP in tribal and backward areas however presented a different perspective [37]. It flagged a poor detection rate also known as a case detection rate (CDR) among tribal and backward areas. It compared the data with other districts in India, which are more developed. The central TB division of the Government of India published the data. Although every patient is monitored and tracked for the treatment purpose throughout the lifecycle of the disease. Twenty-six percentage of the tribally populated districts in India had CDR less than 51 percentage. The study was done in 2012. More than 50 percent of the tribal districts were not able to achieve a full cure rate and stood at less than 85 percent. Only half of the tribal and backward districts were able to achieve the 85 percent cure mark. RNTCP is mainly monitored by statistics like new smear-positive treatment success rate (NSP) and NSP case detection rate. The study also suggested a change in service delivery methodology to encompass completely affected areas.

TB is highly influenced by the company of other chronic diseases like diabetes mellitus. In fact, Diabetes is the risk factor for the occurrence of TB. India has both patients of consumption illness and of diabetes mellitus and they both are on the rise. In this study, 550 confirmed TB patients were taken into account to study the TB and diabetes correlation under RNTCP. Around 15.4 percent of the studied population obtained fatal clinical outcomes. Patients with TB need to be screened for diabetes mellitus as well so that the chance of survival is increased for the TB patient. Diabetes creates biochemical complications, which makes it difficult to fully utilize the anti-TB drugs resulting in slower recovery of the patient [38].

How COVID-19 hampered the control efforts

COVID-19 has unleashed an unprecedented type of tragedy on all of humanity. Millions of people have lost their lives owing to the clinical complications created by COVID-19. All the resources were diverted to the containment of the COVID-19. All the other outpatient departments practically stopped functioning, as the sole focus was to contain the menace of COVID-19 anyhow. One of the campaigns that was doing well and was right track was the TB control programs all over the world. According to the data gathered by the WHO suggested that 1.4 million people were not able to access TB-related healthcare services [39-41]. Access to the TB control program was down by 21 percent, which primarily affects the patients affected in the lowest rung of the socioeconomic strata. 500 thousand additional mortalities are predicted from TB complications if the access is still far from the needy patients. TB is still one of the top mortalities causing infectious disease and the pandemic made it even worse. The WHO and health agencies around the world are coming together along with non-governmental organizations to get back together to eliminate TB anyhow. The effect that COVID-19 has affected not only affected the COVID-19 affected individuals but non-COVID-19 comorbid patients are on the same footing when it comes to the impact of COVID-19. In the reports published by the WHO, India ranks fourth in lack of access to TB health care services and a quarter of the affected individuals get lesser access to care services [42]. A novel approach suggested by the report, which is essential in order to trace the patients without investing extra resources, is testing both COVID-19 and TB suspects at the same time. It will ensure timely detection and we can prevent the increase in mortalities due to late detection. Tracing of households is essential as they are at high risk both for COVID-19 and for TB.

Challenges associated with RNTCP

Although the RNTCP in India has achieved great progress in the fight against TB, it still faces a number of obstacles in its quest to eradicate and control the illness. Drug-resistant strains are becoming more prevalent, and two major challenges are the rise of multidrug-resistant (MDR-TB) and extensively drug-resistant (XDR-TB) TB. More involved and lengthy medication regimens are needed to treat various types of tuberculosis, and they can be expensive and have greater treatment failure rates. Underreporting and underdiagnosis of tuberculosis cases are caused in part by delayed diagnosis and subpar testing techniques. For early detection and prompt treatment start, particularly in distant or resource-constrained places, better and faster diagnostic technologies are required. Many cultures still stigmatize tuberculosis, which makes it difficult for patients to get treatment and stick with it. Successful TB control depends on addressing socioeconomic determinants of health, increasing knowledge, and lowering stigma. TB frequently coexists with other medical conditions such as diabetes, malnourishment, and HIV/AIDS. Specific interventions are necessary for vulnerable populations, such as prisoners, migrants, and people living in cramped or underprivileged environments, as they are more vulnerable.

## Conclusions

TB is certainly a chronic illness that can have a detrimental impact on the person affected. It also impacts socioeconomic conditions as the fallout impacts, and the medications can consume sufficient money. In addition, it has been seen that the prevalence of TB has been among the socioeconomically backward communities and tribal areas where access to healthcare services is low. Even RNTCP has not been able to 100 per cent cover the tribal and backward areas. The impact of COVID-19 on the TB control program has not been assessed, but it sure has a detrimental impact on it. The underlying chronic medical illnesses like diabetes and HIV are huge risks for TB to obtain fatal clinical outcomes. Under the DOTS strategy, more focus must now be on the nutritional needs of the TB patient as immune system building can deter the mycobacterium tuberculosis from worsening further.
